# EXPERIENCES OF OLDER PEOPLE AND INFORMAL CAREGIVERS WITH LIVE-IN MIGRANT CARE WORKERS IN BELGIUM

**DOI:** 10.1093/geroni/igz038.2142

**Published:** 2019-11-08

**Authors:** Sylvia Hoens, An-Sofie Smetcoren, Liesbeth De Donder

**Affiliations:** 1 Vrije Universiteit Brussel, Brussels, Belgium; 2 Vrije Universiteit Brussel, Brussels, Brussels Hoofdstedelijk Gewest, Belgium

## Abstract

The increasing number of older people has a significant impact on the organization of care in European countries. Despite the availability of formal care services, adequate solutions are still missing and older people themselves search for alternative strategies to meet their care needs. For example, a recent tendency is to call upon help of migrant care workers. In Belgium, research concerning this often invisible care solution remains absent. Therefore, the study at hand explores the experiences of the older care users and their informal caregivers and examines how this care can increase their well-being. Eight in-depth interviews with older people who rely on live-in migrant care workers, five interviews with professionals and one focus group with experts have been conducted. This study found that live-in caregivers relieve the informal carers, guarantee the presence of permanent care 24/7 and enable older people to live longer at home.

